# Conceptualising good mental health for people with intellectual disabilities: An inclusive delphi study

**DOI:** 10.1016/j.ijchp.2025.100601

**Published:** 2025-06-28

**Authors:** Sophie Komenda-Schned, Sarah Jasmin Landskron, Paula Moritz, Nicole Braunstein, Josef Hochmeister, Karin Riegler, Robert Saugspier, Brigitte Lueger-Schuster, Luis Salvador-Carulla, Elisabeth Lucia Zeilinger

**Affiliations:** a Department of Clinical and Health Psychology, Faculty of Psychology, University of Vienna, Vienna, Austria; b Vienna Doctoral School in Cognition, Behaviour and Neuroscience, University of Vienna, Vienna, Austria; c Research Institute for Developmental Medicine, Johannes Kepler University Linz, Linz, Austria; d Bundesverband der Lebenshilfe Österreich, Vienna, Australia; e Health Research Institute, Faculty of Health, University of Canberra, ACT, Australia; f Department of Clinical Research SBG, Academy for Ageing Research, Haus der Barmherzigkeit, Vienna, Austria

**Keywords:** Intellectual disabilities, Good mental health, Conceptualisation, Delphi study, Participatory research, Mental Health Experts, Co-Researchers

## Abstract

**Background:**

Currently, there is no conceptualisation of good mental health for people with intellectual disabilities (ID). To develop an initial shared understanding of good mental health in this population, an inclusive Delphi study with two survey rounds was conducted.

**Methods:**

The Delphi study comprised a total of *N* = 60 participants in the first round, and 53 in the second round. In sum, 23 experts with ID and 37 mental health experts were included. The Delphi questionnaire applied a universal design, maximising accessibility for experts with and without ID. Participants were asked to rate the relevance of each item for good mental health of people with ID. People with ID served as co-researchers throughout the research process.

**Results:**

In both survey rounds, all items were rated as important for good mental health of people with ID (weighted median ≥ 3 out of 5). In a structural synthesis, the following factors were found to be important: (1) being part of the community, (2) adequate support, (3) social contacts, (4) communication, (5) working and living environment, (6) keeping the body healthy, (7) no mental disorders, (8) healthcare, (9) psychosocial functioning. As the broadest theme, psychosocial functioning included six subthemes, such as emotions, autonomy and self-concept, and doing something meaningful.

**Conclusions:**

This study provides a foundational step towards developing a more inclusive understanding of good mental health for people with ID. The active involvement of co-researchers underscores the value of participatory methods in shaping research outcomes.

**Accessible Summary**

**Background**

• There is only little research on good mental health of people with learning disabilities.^1^
• In previous studies,
• people with learning disabilities and mental health experts had different opinions on what’s important for good mental health of people with learning disabilities.
• We want to develop an understanding of good mental health which is shared by people with and without learning disabilities.

**Methods**

• A Delphi study is a way to reach agreement between different opinions. We conducted a Delphi study which included 2 groups of experts:
  ○ 37 mental health professionals
  ○ and 23 people with learning disabilities.
• Both expert groups filled in the same survey.
• People with learning disabilities supported this study as co-researchers.

**Results**

• The experts said that the following aspects are important for good mental health in people with learning disabilities:

1. being part of the community
2. needs-oriented support
3. social contacts
4. communication
5. working and living environment
6. keeping the body healthy
7. no mental disorders
8. healthcare
9. psychosocial functioning,for example emotions, autonomy and self-concept, doing something meaningful

**Conclusions**

• This study is a first step towards an understanding of good mental health which is shared by experts with and without learning disabilities.
• Additionally, this study is a good example of doing research with co-researchers with learning disabilities.

## Introduction

Intellectual disabilities (ID) are diagnosed when a person’s intellectual and adaptive abilities are limited significantly (including IQ < 70). These disorders are categorised into four levels—mild, moderate, severe, and profound—based on the degree of impairment in intellectual functions and adaptive behaviour (American Psychiatric Association, 2013; World Health Organization, 2018).

Despite their ID people with ID can be mentally ill or mentally healthy, just like any other person. They are more prone to developing mental disorders (Cooper et al., 2015), due to biological causes as well as elevated exposure to risk factors, like abuse and trauma (Rittmannsberger et al., 2019; Wigham & Emerson, 2015). While there is a body of research on mental disorders and treatment in this population (Cooper et al., 2007, 2015; Griebler et al., 2021; Munir, 2016), research on prevention and mental health promotion is scarce (Perry et al., 2010; Razza & Tomasulo, 2005).

According to medical models of disabilities (American Psychiatric Association, 2013; World Health Organization, 2018), ID are a psychiatric diagnosis which impact a person´s mental health by definition. Person-centred integrative diagnosis is discussed to be a relevant approach to describing the health status of people with ID in a holistic way, as it includes the health condition and disability but also wellbeing and positive functioning (Bertelli et al., 2021; Mezzich et al., 2010). In the current scientific discourse, good mental health is discussed to be a multidimensional concept that is correlated, yet distinct to mental illness (Keyes, 2005). One of the most popular definitions is the following: Good mental health is *“a state of well-being in which every individual realizes his or her own potential, can cope with the normal stresses of life, can work productively and fruitfully, and is able to make a contribution to her or his community”* (World Health Organization, 2004). However, this definition has been developed for the general population. While there is growing research on good mental health of other marginalised groups (Ali et al., 2023; Johansson et al., 2007; Lyons et al., 2013), the topic has not yet been studied in people with ID (Komenda-Schned, Landskron, et al., 2024). It remains unclear whether adjustments to this definition would be necessary for people with ID to address their functional limitations in daily life due to their ID.

Despite numerous studies in positive psychology and ID on aspects such as wellbeing or quality of life (Albaum et al., 2021; Keyes, 2005; Komenda-Schned, Landskron, et al., 2024), there is little research on good mental health in this population (Bailey et al., 2022; Bertelli et al., 2021; Komenda-Schned, Landskron, et al., 2024; Moritz et al., 2024). A recent systematic review found no ID-specific definition of good mental (Komenda-Schned, Landskron, et al., 2024). Without a comprehensive definition of this concept, health disadvantages for people with ID will likely persist, as, for instance, the development of population-specific, evidence-based mental health promotion programs is hindered (Fledderus et al., 2010; Jané-Llopis & Barry, 2005). This indicates non-compliance with Article 25 of the Convention on the Rights of Persons with Disabilities (UN—CRPD; United Nations, 2006), which advocates for “ the same range, quality and standard of […] healthcare and […] population-based public health programs” for people with ID and the “highest attainable standard of health without discrimination on the basis of disability” (United Nations, 2006). High-quality mental health programs are urgently needed for this population to promote good mental health and reduce the increased risks of developing mental disorders among people with ID (Cooper et al., 2007, 2015; Griebler et al., 2021; Munir, 2016).

To develop a shared understanding of good mental health in people with ID, triangulation of data from mental health experts, caregivers and people with ID themselves is crucial. Mental health experts offer important perspectives based on their scientific and clinical experiences (Meuser & Nagel, 2005), while caregivers and people with ID provide authentic insights into health-maintaining and -promoting factors within the daily lives of people with ID (van Herwaarden et al., 2022). Involving multiple participant groups enhances the richness and depth of the data, facilitating a more thorough understanding of the construct of interest (Donkoh & Mensah, 2023; Heale & Forbes, 2013). This approach has been particularly beneficial in under-researched fields within the ID population (van Herwaarden et al., 2022).

To explore the topic of good mental health in people with ID a participatory, multi-stage research project has been implemented, including expert interviews with mental health professionals, focus groups with people with ID, and a systematic review (Komenda-Schned et al., 2024, Komenda-Schned et al., 2025, Komenda-Schned et al., 2025; Komenda-Schned et al., 2025). The different perspectives on mental health that were identified formed the basis for the design of the Delphi study, which is an effective tool for integrating diverse viewpoints into a coherent, well-founded agreement, particularly in areas where research is limited. It allows for anonymous, structured communication among experts in a multi-stage process, which facilitates equal consideration of all perspectives and leads to deeper insights by combining individual expertise into cumulative knowledge (Linstone & Turoff, 2002). Although rarely used, the Delphi method has been proven to effectively include experts with and without ID in the same panel, provided that adaptations in survey accessibility are made (Piantedosi & O’Shea, 2023).

This study’s primary aim was to develop a set of constituting factors of good mental health in people with ID, including mental health experts and experts with ID in the same Delphi survey. To achieve this, an inclusive Delphi framework was established.

## Methods

This study has been pre-registered via OSF Registries (Komenda-Schned, Moritz, et al., 2024) and it has been approved by the Ethics Committee of the study site (No. 00885). This study applied a participatory research approach. People with ID acted as co-researchers and were involved in every step of the research process.

### Participatory research process

This study was conducted in close partnership with four co-researchers with ID (NB, JH, KR, RS), who actively participated throughout the whole research process. Their roles included (1) testing the rating scale, (2) assessing the comprehensibility of the items, (3) piloting the Delphi survey, (4) supporting the final data analysis, and (5) assisting in the dissemination of the study’s findings. Team meetings were held regularly every four to eight weeks, either online or in person. Communication occurred on an equal footing, allowing all (co-)researchers to contribute their full range of competencies.

In participatory research the researchers’ academic knowledge and the co-researchers’ life experiences are considered equally valuable (Buck et al., 2024; Munde & Tillmann, 2022; Salvador-Carulla et al., 2017; Sullivan et al., 2018; Walmsley et al., 2018). This approach heightens the chances of identifying the true needs of the target population (Di Lorito et al., 2017; Walmsley et al., 2018). Furthermore, it facilitates the study conduction through easily accessible study materials, empowers people with ID (Munde & Tillmann, 2022; Walmsley et al., 2018), and it enriches the academic researchers as well as the involved co-researchers. Moreover, participatory research is in line with the UN—CRPD (United Nations, 2006) which emphasises the “full and effective participation and inclusion” of people with ID.

### Participants

The Delphi study included a total of *N* = 60 participants. Two groups of experts participated in the Delphi study: (1) international mental health professionals or professional caregivers with expertise in ID who work either scientifically or practically in this field, and (2) people with ID, who are inherently experts on their own account regarding their mental health. Mental health experts and professional caregivers offer meaningful perspectives based on their professional knowledge and experiences in scientific and clinical environments (Meuser & Nagel, 2005), whereas people with ID provide valuable insights into their mental health needs (Havercamp et al., 2022). In the first round, *n* = 23 experts with ID and *n* = 37 mental health experts participated, following a total of *N* = 53 participants in the second round, including *n* = 21 experts with ID and *n* = 32 mental health experts. Seven participants dropped out (*n* = 2 people with ID, *n* = 5 mental health experts). Details on participant’s sociodemographic data are depicted in Table 1.

**Table 1:** Sociodemographic data of experts with and without ID in the first survey round.

	**Experts with ID (*n* = 23)**	**Experts without ID (*n* = 37)**
**Gender**	12 female, 11 male	26 female, 11 male
**Age**	23–63 years (*M* = 43.35, *SD* = 14.05)	27–73 years (*M* = 48.19, *SD* = 12.68)
**Nationality**	AT = 18 DE = 2 CH = 3	AT = 14 DE = 14 CH = 6 NL = 1 UK = 1 IT = 1
**Occupation / Profession**	1st labour market: *n* = 3 2nd labour market: *n* = 4 3rd labour market: *n* = 14 not specified: *n* = 2	nurses: *n* = 5 psychologists / psychotherapists: *n* = 11 physicians: *n* = 14 caregivers: *n* = 7
**Living situation**	supported living facility: *n* = 7 with family: *n* = 2 own appartement: with support: *n* = 9 without support: *n* = 5	

*Note*. Of the experts with ID *n* = 10 stated to be self-advocates. Nationalities: Austria (AT), Germany (DE), Switzerland (CH), Netherlands (NL), United Kingdom (UK), Italy (IT). Occupation: 1st labour market (regular employment), 2nd labour market (integrative or supported employment), 3rd (sheltered workshop).

### Materials and measures

#### Delphi survey

The item pool for the Delphi survey was based on triangulated data originating from expert interviews with mental health professionals, focus groups with people with ID, and a systematic literature review (Komenda-Schned et al., 2024, Komenda-Schned et al., 2025, Komenda-Schned et al., 2025).The online survey presented 60 items in the first round, and 67 items in the second round. We organized the items into the following 13 content-related pages to provide a clear structure that would make the questionnaire easier to complete and less overwhelming for participants: (1) work, (2) living, (3) social contacts, (4) responding well in social situations, (5) competencies, (6) leisure, (7) appropriate support, (8) my feelings, (9) self-confidence/self-concept, (10) having energy and being able to relax, (11) keeping the body healthy, (12) mental disorders, (13) being part of the community. The headings of the individual survey pages were chosen in collaboration with our co-researchers to ensure accessibility. The full list of items is provided in supplementary file A, including information on the source from which each item was derived.

Participants were asked to rate the importance of each aspect regarding its contribution to good mental health of people with ID in two steps: First, they were asked to rate whether the aspect was important for good mental health (visual rating scale: green tick or red cross). Second, if an aspect was judged to be important, the importance of the aspect had to be rated on a visual rating scale ranging from one to five stars (i.e., one star = *a* little important, two stars = rather important, three stars = important, four stars = very important, five stars = very-very-important). If an invalid answer combination (e.g., red cross and stars) was selected, participants received an error message. Supplementary file B shows a flowchart of the Delphi survey.

**Fig. 1:**
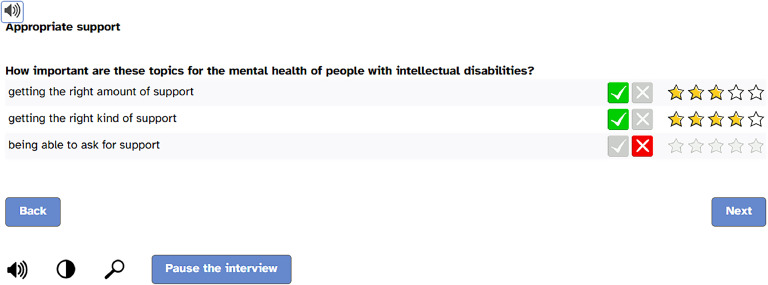
Screenshot of the online survey.

The survey applied a universal design (Meyers & Andresen, 2000) using easy-to-read language and a screen-reader function to be as accessible as possible and applicable for both expert groups. Fig. 1 displays a screenshot of the online survey. On the last page of the questionnaire, participants could add new items in an open text field if they felt that an important aspect of good mental health was missing. For the second Delphi round, the questionnaire was adapted according to the item ratings obtained in the first round (see 2.5. Data analysis). The response mode for the second round remained unchanged. Additionally, participants were given feedback on the average ratings of each item in the first round and new items were highlighted in colour.

#### Usability-survey for mental health experts

To assess the acceptance of the universal design, a usability questionnaire was implemented in the first round of the survey for mental health experts. It comprised nine items (e.g. *The individual pages of the survey appear clear and well structured.*) rated on a 5-point Likert scale to evaluate participants' experiences. In advance, the usability assessment was piloted to ensure feasibility.

### Sampling strategy

Experts with ID were recruited via national support organisations for people with ID in Austria. To cover a heterogeneous sample regarding the degree of ID, age, living and working situation, support providers with different service offerings were contacted in a targeted manner. However, due to the high cognitive demands of the study design and self-selection, we expected most of the sample to have mild to moderate ID. The international mental health experts were recruited via organisations that address the scientific and practical work for and with people with ID and via a snowball procedure. Additionally, national experts were recruited via known experts in the field (e.g. psychologists/psychiatrists working with people with ID) and their contacts.

The contact details of all participants, collected and stored separately from the completed survey data from the first round, were used to invite participants to participate in the second round. Individual links were provided to ensure that each participant completed the second survey.

### Procedure

**Fig. 2:**
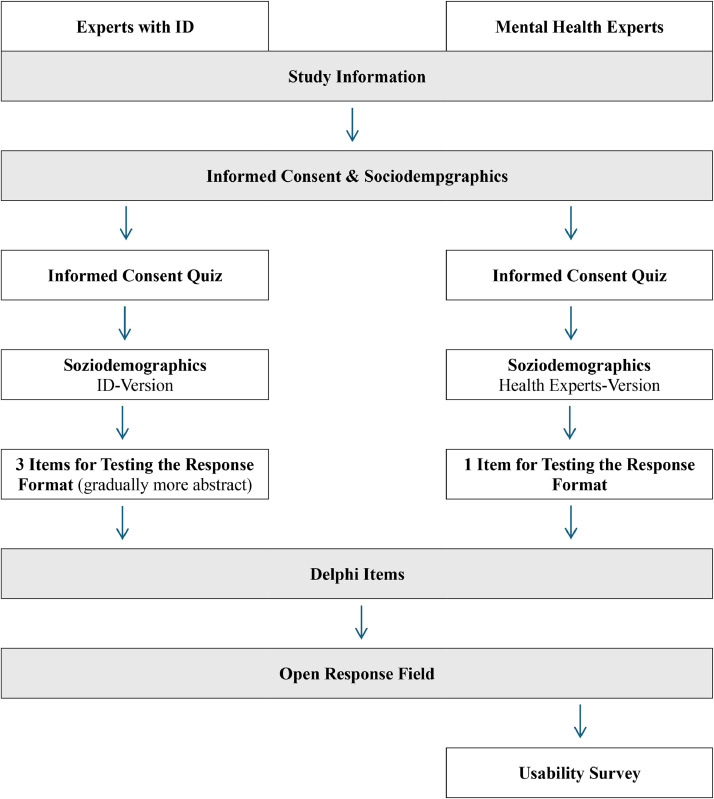
Survey sequence for experts with and without an ID in the first survey round.

Data was collected in two consecutive Delphi rounds to reach consensus on relevant aspects of good mental health of people with ID. The first round was conducted from June to August 2024; the second round from October to December 2024. To obtain informed consent and ensure the understanding of the study information for all participants, a short quiz was presented, which covered the topics of voluntariness, anonymity and the necessity of the second survey round. The minor differences in the survey of experts with and without ID are shown in Fig. 2. The survey of the second round was identical for experts with and without ID. Data collection was stopped after the second Delphi round, to prevent high drop-out rate and participation fatigue (Linstone & Turoff, 2002; Vicente et al., 2019).

To reach (inter-)national experts with and without ID and to reduce bias due to population areas (e.g. rural, urban), the Delphi survey was implemented as an online questionnaire via SoSci Survey (SoSci Survey GmbH, 2024). Experts with ID were supported by a caregiver to complete the online survey, if necessary. To reduce (interviewer) bias, the caregivers were provided with a manual to enable structured and neutral guidance throughout the survey. The manual was developed in close collaboration with two caregivers and is provided in supplementary file C.

### Data analysis

Data analysis was conducted using SPSS (v. 29.0.1.0 IBM Corp., 2013). After data preparation, weighted medians were calculated for all items to account for differences in sample size. This ensured that the views of both expert groups were included with the same value and significance. Given the verbal categorisation of items as *important*, ratings with a median of three or more stars were categorised as approval, while those with a median of less than three stars were classified as disagreement and excluded from further analysis. Following a qualitative framework (Mayring & Fenzl, 2019), the open-ended responses of the first survey round were analysed to identify new items.

To obtain a structural synthesis of the relevant aspects of mental health in people with ID, all items were clustered after the completion of the second Delphi round. In a collaborative process, using a puzzle analogy, the co-researchers (NB, JH, KR, RS) contributed to organising, defining, and naming the final themes. Consequently, the final themes are formulated in easy-to-read language, even when representing psychological constructs (e.g., social competence is described as responding well in social situations). In the last step, the items corresponding to the final themes were combined to scales of which weighted descriptive statistics were computed. Potential differences between both groups of experts were explored using unweighted descriptive statistics.

## Results

### First round

Weighted medians of all items ranged from 2.84 to 5.00. After rounding to integer values all items had weighted medians of 3 or above (e.g. important, very important, or very-very important), indicating that no item had to be eliminated for the second survey round. Even though all items were widely approved by the participants of both groups, ceiling effects could be observed for experts with ID (73.33 % of all items had medians of 5.00, and 96.67 % medians of 4.00 or 5.00, as opposed to 23.33 % and 78.33 % in the experts without ID-group). Items with the lowest ratings are displayed in Table 2.

**Table 2:** Lowest rated items of the first survey round.

Item	Joint analysis (Mdn_weighted_)	Experts with ID (Mdn)	Experts without ID (Mdn)
To believe in something (e.g. going to church).	2.84	3.00	2.00
Not showing behaviour that disturbs others.	3.00	4.00	2.00
Basic competencies (e.g. reading, writing, calculating).	3.00	5.00	2.00
Learning something about health.	3.00	3.00	3.00

According to the responses given by participants in the open text fields of the first survey round, the items displayed in Table 3 were added to the second survey round. Moreover, minor *amendments* were made to three items, as displayed in Table 3 as well.

**Table 3:** New and amended items in the second survey round.

**New items in the second survey round**
Being allowed to choose one’s own living situation (e.g. moving out of the parent’s home or picking the residential area).
Having a good daily routine (e.g. getting up at a certain time, having coffee in the afternoon).
Having doctors and therapists who are well informed about people with intellectual disabilities.
It should be checked if doctors and therapists are working correctly (e.g. quality control).
Being able to communicate with the people around you.
Others understand how someone speaks (e.g. with a talker).
Being able to understand what others are saying.
**Amendments to existing items in the second survey round**
Being able to live out one’s sexuality (e.g. being touched gently, having sex, *masturbation*).
Learning something about health *(*e.g. *attending courses)*.
Being respected by others *(*e.g. *others take people with intellectual disabilities seriously, people with intellectual disabilities do not have to be ashamed or hide from others)*.

*Note.* The parts written in *italics* were added.

### Second round

Weighted medians of all items ranged from 3.00 to 5.00 (ratings from *important* to *very-very-important*). No item had to be removed, and no new items were proposed by the participants, indicating the relevance and comprehensiveness of the item set. In the group of experts with ID, high ceiling effects were observed (88.06 % of all items had medians of 5.00, and 98.51 % had medians of 4.00 or 5.00, as opposed to 31.34 % and 82.09 % in the experts without ID-group). The items with the lowest ratings (weighted medians of 3.00) remained the same as in the first round (see Table 3).

### Structural synthesis: good mental health of people with ID

**Fig. 3:**
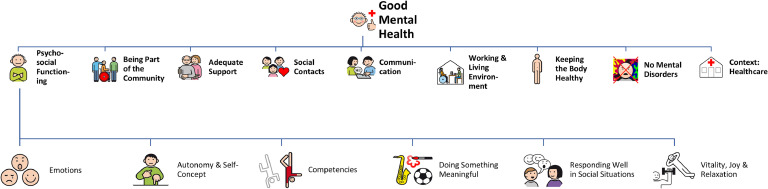
Constituting factors of good mental health of people with ID.

After clustering the items of the second survey round together with the co-researchers with ID, the final scales were formed accordingly. The final scales and sub-scales are displayed in Fig. 3.

#### Being part of the community

This scale encompasses different dimensions of inclusion, such as person-environment-fit, and overall accessibility. Additionally, it includes social participation, such as contributing to the community (e.g. being involved in the neighbourhood or in a club).

#### Adequate support

Adequate support emphasises both the type and extent of personal assistance in everyday life, which must be tailored to an individual’s specific needs. Similarly, seeking support in this context is also encompassed within this theme.

#### Social contacts

On a broader level, social contacts involve feeling respected and being taken seriously by others. More specifically, a distinction can be drawn between feeling connected with other people (e.g. colleagues or caregivers) and emotional closeness, which encompasses close relationships, such as those with family, close friends, or a romantic partner. Expressing one´s sexuality, including caresses and masturbation, is also part of this scale.

#### Communication

Communication includes aspects of expressive as well as receptive language. The key element is understanding and being understood by other people, independent of the mode of communication (e.g. verbal, non-verbal, assisted and augmented communication).

#### Working and living environment

The scale consists of aspects related to both one’s living situation and occupational environment, with a focus on maintaining a regular daily routine. In terms of work, the scale covers having a job, such as in a sheltered workshop or in a coffee-house, being satisfied (e.g. having an interesting work task or having little stress), and feeling comfortable at work (e.g. getting along well with colleagues, looking for each other). For living, it includes having privacy and the possibility of being alone, feeling safe and comfortable at home (e.g. getting along well with roommates, having a nice room), and being allowed to choose one’s own living situation (e.g. moving out of the parent’s house or picking the residential area).

#### Keeping the body healthy

This scale includes individual physical aspects of good mental health, like healthy food, movement and exercise and good sleep. Health behaviours, like actively doing regular check-ups, and not experiencing pain, are also part of keeping the body healthy.

#### No mental disorders

No mental disorders include the absence of psychiatric symptoms, psychological difficulties as well as challenging behaviour.

#### Healthcare

The contextual topic of healthcare comprises the aspect of access to the healthcare system, e.g. treatment of mental health issues, and high quality of provided services in the broader societal and systemic context. This is reflected in having physicians and therapists who are experienced with people with ID as well as in quality assurance measures.

#### Psychosocial functioning

Psychosocial functioning is the richest and most comprehensive scale and was therefore divided into six subscales: (i) vitality, joy, and relaxation, (ii) emotions, (iii) autonomy and self-concept, (iv) responding well in social situations, (v) doing something meaningful, and (vi) competencies.

**Vitality, joy, and relaxation.** This scale is split up into two categories (i) joy and vitality and (ii) relaxation. The former refers to having energy, being motivated and satisfied with one´s life. Having fun and laughing as well as a basic optimism are also part of this aspect. Relaxation describes a state of tranquillity and the absence of stress. This includes taking time for oneself and clearing one´s mind.

**Emotions.** Emotions include affective states, such as being in a good mood or feeling a variety of emotions. It also refers to the aspects of emotional competence (e.g. *noticing how I feel*) and emotion regulation (e.g. *being able to deal with my feelings*).

**Autonomy and self-concept.** Autonomy and self-concept describe aspects of self-determination, independence, self-confidence and self-efficacy. This includes making your own decisions, having confidence in your abilities and knowing what is good for you. Another aspect is being able to be yourself, which also includes standing up for yourself and setting boundaries. Personal growth is a further component of this scale.

**Responding well in social situations.** Responding well in social situations means solving problems and conflicts with others, getting along well with others, being able to take the perspectives of others, and supporting others.

**Doing something meaningful.** Doing something meaningful involves engaging in activities that are important to oneself, such as leisure activities and hobbies (e.g. being out in the fresh air, going on vacation). It also includes to believe in something (e.g. going to church).

**Competencies.** The scale comprises developing basic competencies like reading, writing, and calculating. It also covers the ability to use public transport and going somewhere independently (e.g. by bus). Further aspects are taking care of daily life tasks, such as grocery shopping, cleaning, and cooking, as well as reaching own goals. Additionally, learning about health is a component of competencies.

### Consensus among the experts

Table 4 displays the weighted descriptive statistics (joint analysis) for all scales after the second round of the Delphi survey. The scales are arranged from the lowest to the highest ratings of both expert groups (according to the results of the joint analysis). Moreover, the analysis for each group is shown separately. Meaningful differences (highlighted in bold numbers) between the ratings of the mental health experts and the experts with ID occurred in the scales *Competencies* and *Responding well in social situations*. In both cases the mental health experts rated the scales‘ relevance for good mental health significantly lower than the experts with ID. In all other scales the mean ratings of experts with and without ID differed by less than 0.45, indicating high levels of consensus between the two groups.

**Table 4:** Descriptive statistics for all clusters in the joint analysis as well as the separate expert groups.

	**Joint analysis**		**Mental health experts**		**Experts with ID**	
**Scale**	**Mean (Std)**	**IQR**	**Mean (Std)**	**IQR**	**Mean (Std)**	**IQR**
Competencies	3.57 (1.09)	1.20	**3.02** (0.90)	1.35	**4.12** (0.98)	1.10
Doing something meaningful	3.59 (0.89)	0.67	3.43 (0.74)	0.92	3.76 (1.01)	1.33
No mental disorders	3.69 (1.11)	1.33	3.50 (0.99)	1.00	3.88 (1.22)	1.58
Responding well in social situations	3.87 (1.05)	1.13	**3.53** (0.85)	0.94	**4.21** (1.13)	1.13
Emotions	3.91 (0.84)	0.88	3.70 (0.74)	0.94	4.13 (0.90)	1.13
Being part of the community	4.00 (0.93)	1.00	3.94 (0.58)	0.50	4.06 (1.21)	1.63
Vitality, joy, and relaxation	4.04 (0.87)	1.38	3.84 (0.65)	1.00	4.24 (1.02)	0.94
Keeping the body healthy	4.08 (0.83)	0.80	4.12 (0.71)	0.80	4.03 (0.95)	0.95
Autonomy and self-concept	4.13 (0.80)	1.11	3.92 (0.70)	0.86	4.34 (0.86)	1.17
Healthcare	4.24 (0.86)	1.33	4.39 (0.80)	0.92	4.08 (0.90)	1.67
Social contacts	4.24 (0.92)	0.88	4.34 (0.62)	0.50	4.13 (1.16)	1.38
Communication	4.36 (0.95)	0.79	4.52 (0.51)	0.67	4.18 (1.24)	1.00
Working and living environment	4.38 (0.58)	0.50	4.42 (0.34)	0.44	4.35 (0.76)	0.94
Adequate support	4.53 (0.81)	0.67	4.67 (0.49)	0.67	4.40 (1.03)	0.83

On item level, several items could be identified where the ratings of the two groups differed by 2.00 in medians. These items and their medians in each group are displayed in Table 5.

**Table 5:** Largest group differences in medians for single items.

**Scale**	**Item**	**Mental health experts (Mdn)**	**Experts with ID (Mdn)**
Responding well in social Situations	• Being able to take the perspective of others.	3.00	5.00
	• Supporting others (e.g. person with ID can support somebody else).	3.00	5.00
Competencies	• Basic competencies (e.g. reading, writing, calculating).	2.00	4.00
	• Being able to go somewhere independently (e.g. by bus).	3.00	5.00
	• Taking care of daily life tasks (e.g. grocery shopping, cleaning, cooking).	3.00	5.00
Autonomy and self-concept	• Being independent.	3.00	5.00
	• Personal growth (e.g. learning from mistakes, trying something new).	3.00	5.00
Vitality, joy and relaxation	• Being optimistic (e.g. always seeing the positive).	3.00	5.00
Being part of the community	• Making a contribution to the community (e.g. campaigning for an important issue).	3.00	5.00

### Usability-Survey

95 % of the mental health experts (*n* = 35 of 37) completed the usability survey. A detailed summary of the findings is presented in supplementary file D.

## Discussion

This study presents a newly identified set of constituting factors of good mental health in people with ID, comprising eight main themes: (1) Being Part of the Community, (2) Adequate Support, (3) Social Contacts, (4) Communication, (5) Working and Living Environment, (6) Keeping the Body Healthy, (7) No Mental Disorders, and (8) Psychosocial Functioning, which encompasses six subthemes: (i) vitality, joy, and relaxation, (ii) emotions, (iii) autonomy and self-concept, (iv) responding well in social situations, (v) doing something meaningful, and (vi) competencies.

Moreover, the contextual theme of healthcare was identified, which includes healthpolitical aspects, like equal access to high-quality healthcare for people with ID or specific education and training for health professionals. This finding concurs with prior research, which identified the absence of adequate services as well as organisational factors and substantial knowledge gaps of health care personnel as major barriers for accessible healthcare for people with ID (Whittle et al., 2018). This contextual theme addresses a relevant political framework that, if realised, could substantially facilitate mental health care for people with ID. Since people with ID and their caregivers can hardly impact this framework directly, these aspects are considered as relevant contextual dimensions.

When comparing our set of factors of good mental health with the WHO’s definition (2004) of the construct, all aspects of good mental health, are mirrored by our findings, with the exception of “working productively and fruitfully”. For people with ID, productivity at the workplace does not impact their mental health, as this aspect did not emerge in the explorative studies on which the Delphi item set was grounded (Komenda-Schned et al., 2025, Komenda-Schned et al., 2025; Komenda-Schned, Landskron, et al., 2024), while aspects like having and liking a job or feeling comfortable at work seem to be much more important for good mental health of this population. However, the identified set of factors of good mental health for people with ID also goes beyond the WHO’s definition. The themes adequate support, a suitable working and living environment, social contacts and communication are not mentioned in the WHO’s definition, though they represent the four highest rated themes, according to our expert panel.

Existing research emphasised the importance of these factors for promoting mental health for people with ID across various contexts. In previous studies, people with ID reported higher levels of subjective wellbeing, which is considered to be an integral part of good mental health (World Health Organization, 2004), when maintaining regular contacts with peers (Emerson & Hatton, 2008). Moreover, positive and close social relations were associated with higher levels of social integration (Nota et al., 2007), prevented loneliness, were protective against the development of mental disorders (Lunsky & Benson, 2001; Meins, 1993; Scott & Havercamp, 2014), and fostered good mental health (Kawachi & Berkman, 2001; Siedlecki et al., 2014). In contrast, adults with ID who experienced communication difficulties had a higher risk of social isolation (Smith et al., 2020). Severe levels of ID, reduced social participation, and challenging behaviour were the main predictors of communication difficulties, which, in turn, inhibited social integration (Smith et al., 2020). These findings are in line with our results and highlight both the relevance of communication and social contacts in supporting good mental health.

Another factor that was among the most important ones, according to the expert panel, was adequate support. This finding is in line with previous research, which found that for people with ID, staff members often represented one of the primary social contacts (Lippold & Burns, 2009), making a significant contribution to providing essential support. In this regard, when staff members focused on how people with ID prefer to communicate, it improved their self-determination (Vicente Sánchez et al., 2022), their participation in decisions about their support arrangements, and enhanced the adequacy and quality of support (Lutz & Fisher, 2023). Moreover, studies showed that person-centred support which focused on self-determination, resources, skills and abilities of people with ID had empowering effects and improved their wellbeing (Blaise et al., 2025; Salvador-Carulla et al., 2012).

A prominent context for the provision of needs-oriented, individualised support is the working and living environment of people with ID, which offers multiple ways to foster wellbeing and participation (Joyce et al., 2024; Ribenfors et al., 2025; Yong, 2023). In accordance with previous research (Ribenfors et al., 2025; Yong, 2023), the results of this study showed that living environments providing safety and comfort, opportunities for control, choice, and the acquisition of new skills, as well as a sense of belonging supported people with ID’s wellbeing. Additionally, the wellbeing of people with ID was enhanced by workplaces that offered various opportunities and roles, tasks and environments tailored to the person’s capabilities and needs, opportunities for continuous learning, as well as individualised, holistic support (Joyce et al., 2024; Yong, 2023). Adequate support in an inclusive working environment was also associated with elevated levels of job satisfaction in people with ID (Kocman & Weber, 2018), an aspect of overall satisfaction with life (Rojas, 2006). As our study’s results indicate, life satisfaction itself is also an important aspect of good mental health for people with ID. It can be concluded that the themes adequate support, a suitable living and working environment, positive social contacts, and communication are not only highly relevant for good mental health of people with ID, but, as previous research showed (Blaise et al., 2025; see Emerson & Hatton, 2008; Lutz & Fisher, 2023; Nota et al., 2007; Salvador-Carulla et al., 2012; Scott & Havercamp, 2014; Smith et al., 2020; Vicente Sánchez et al., 2022; Yong, 2023), they also seem to be interrelated to some extent (e.g. communication impacts social contacts and integration, suitable working and living environments are places of support provision as well as social integration).

Despite the various homogeneous responses of the two expert groups in our sample, some differences were observed in their ratings: Experts with ID considered their own competencies as well as their social competencies (i.e. responding well in social situations) to be very important for good mental health of people with ID, while experts without ID rated these scales approximately one point lower (in medians). A possible explanation for these differences might be that people with ID experience themselves as competent and are able to use their skills and capabilities, when they find themselves in a supportive living or working environment fitting with their needs. Experts without ID may tend to focus more on the deficits and support needs of people with ID rather than on their competencies and (social) skills that emerge when those support needs are met (Andresen et al., 2001; Chinn, 2014; Geukes et al., 2018).

Moreover, in the context of supported living facilities it was shown, that caregivers encouraged their clients competencies to a larger extent than their autonomy or relatedness (Embregts et al., 2019). This might contribute to the high relevance ratings of competencies and social skills in the group of experts with ID, especially since three quarters of this sub-sample had continuous or occasional support in their living environment. However, the observed discrepancies in the ratings of the two expert groups might also be related to a larger acquiescence bias in experts with ID as opposed to experts without ID (Havercamp et al., 2022), or to the fact that self-report ratings in people with ID tend to be higher than proxy-report ratings, especially regarding wellbeing and mental health assessments (Claes et al., 2012; Cummins, 2002; Santoro et al., 2022; Schmidt et al., 2010; Simões & Santos, 2016).

### Strengths and limitations

Due to uneven sample sizes in the two expert groups, data was weighted to ensure an equal impact of both groups on the results. This procedure could have led to the overrepresentation of outliers. However, this was widely accounted for in our analyses by calculating medians instead of means. In the group of experts with ID ceiling effects were observed, which might originate from acquiescence bias (Havercamp et al., 2022), rather than from high ratings of item importance. This cannot be ruled out completely, because the participants with ID were supported by their caregivers and not by members of the research team. Even though we provided manuals for caregivers, we cannot be certain if the caregivers have read them and followed the guidelines while supporting an expert with ID during the completion of the two surveys. As we used online surveys that relied on verbal information for item presentation, it is most likely that only people with mild or moderate ID are included in the ID expert group and people with limited verbal abilities, severe or profound ID were excluded. The mental health expert group included health professionals as well as professional caregivers. However, family caregivers or relatives of people with ID might also have important perspectives on the topic of good mental health in this population, that are not included in this research. Furthermore, we only collected specific usability feedback from mental health experts, but not from people with ID, due to the already considerable length of the survey. Although the questionnaire was piloted with co-researchers, receiving direct feedback from a broader group of participants with ID could provide valuable additional insights.

It has to be noted that during the two survey rounds seven items were added but due to high relevance ratings of both expert groups no items were excluded from the original item set. Rather than indicating limited convergence we strongly believe this reflects the fact that the items were not generated ad hoc, but based on preliminary studies (e.g. Komenda-Schned et al., 2025; Komenda-Schned, Landskron, et al., 2024) where the respective groups independently identified key aspects of good mental health for people with ID. This extensive groundwork contributed to the high levels of agreement observed in the two Delphi rounds.

To our knowledge, this is the first inclusive Delphi study that used the same survey for experts with and without ID. High rates of approval in the usability survey demonstrate the feasibility and acceptance of such an approach. Moreover, this study shows the relevance and practicability of participatory research settings.

## Conclusions and implications for practice

This study provides a set of constituting factors of good mental health for people with ID. These factors differ from how good mental health is usually defined for the general population. For people with ID, aspects such as a suitable working and living environment, adequate support, communication, and social contacts seem to be more important, while productivity may be less relevant.

As this is the first inclusive Delphi study on comprising factors of good mental health in people with ID, the robustness of its results needs to be evaluated in research and practice. Therefore, the use of other research designs and/or target groups (e.g. family caregivers) would be advisable. Furthermore, the results of this study could serve as a foundation for the development of information campaigns and strategies on the promotion of good mental health among people with ID. This should include the generation and dissemination of easy-to-read materials that are accessible to people with ID. Caregivers and mental health professionals should be informed about relevant aspects of good mental health in people with ID. Mental health promotion activities should ideally take place in a participatory process, together with people with ID, their caregivers, and mental health professionals. Evidence-based mental health promotion programs would contribute to increasing the health equity of people with ID, as claimed by the UN—CRPD (United Nations, 2006), and could significantly contribute to more inclusive and effective public mental health strategies.

## CRediT authorship contribution statement

Sophie Komenda-Schned: Conceptualisation, Methodology, Formal Analysis, Validation, Investigation, Data Curation, Writing – Original Draft, Writing – Review & Editing, Visualisation; Sarah Jasmin Landskron: Methodology, Formal Analysis, Validation, Investigation, Writing – Original Draft, Writing – Review & Editing; Paula Moritz: Methodology, Formal Analysis, Validation, Investigation, Writing – Original Draft, Writing – Review & Editing; Nicole Braunstein: Methodology, Formal Analysis, Writing – Review & Editing; Josef Hochmeister: Methodology, Formal Analysis, Writing – Review & Editing, Karin Riegler: Methodology, Formal Analysis, Writing – Review & Editing, Robert Saugspier: Methodology, Formal Analysis, Writing – Review & Editing, Brigitte Lueger-Schuster: Supervision, Writing – Review & Editing; Luis Salvador-Carulla: Supervision, Writing – Review & Editing; Elisabeth Lucia Zeilinger: Conceptualisation, Methodology, Supervision, Writing – Original Draft, Project Administration, Funding Acquisition

## Data availability statement

Anonymised data of the first and second survey round are available via OSF (https://osf.io/tvpfg/files/osfstorage).

## Declaration of competing interest

The authors declare that they have no known competing financial interests or personal relationships that could have appeared to influence the work reported in this paper.

## Appendix Supplementary materials

Image, application 1

Image, application 2

Image, application 3

Image, application 4

## Footnotes

1 The term learning disabilities is widely used by people with intellectual disabilities to refer to their own group (Schanze, 2017). It is therefore used in the accessible summary. All other parts of this paper use the term intellectual disabilities, in accordance with currently agreed upon scientific terminology.

## References

[bib0001] Albaum C., Chan V., Sellitto T., Vashi N., Hastings R., Weiss J. (2021). Redressing the balance: A systematic review of positive psychology in the intellectual disability literature. International Review of Research in Mental Retardation.

[bib0002] Ali S., Harrichand J.J.S., Shillingford M.A., Herbert L. (2023). A qualitative investigation of Guyanese American perceptions of mental health. The Professional Counselor.

[bib0003] American Psychiatric Association. (2013). *Diagnostic and statistical manual of mental disorders*. Author.

[bib0004] Andresen E.M., Vahle V.J., Lollar D. (2001). Proxy reliability: health-related quality of life (HRQoL) measures for people with disability. Quality of Life Research.

[bib0005] Bailey D.-A., Ford L., Knight V.F. (2022). Exploring perceptions of positive mental health in young adults with intellectual disabilities. Journal of Applied Research in Intellectual Disabilities.

[bib0006] Bertelli M.O., Salerno L., Rondini E., Salvador-Carulla L., Amelung V., Stein V., Suter E., Goodwin N., Nolte E., Balicer R. (2021). Handbook integrated care.

[bib0007] Blaise M., Schroeder M., Suhrcke M., Komenda-Schned S., Zeilinger E.L., Weber G. (2025). Exploring self-determination and satisfaction with life and services of people with disabilities. International Journal of Developmental Disabilities.

[bib0008] Buck A.S., Chapman R., Krahn G.L., Brown C., Gertz B., Havercamp S.M., The Ohio State University Nisonger RRTC on Health and Function (2024). Research about us, with us: an inclusive Research case study. Intellectual and Developmental Disabilities.

[bib0009] Chinn D. (2014). Critical health literacy health promotion and people with intellectual disabilities. Asia-Pacific Journal of Health, Sport and Physical Education.

[bib0010] Claes C., Vandevelde S., Van Hove G., van Loon J., Verschelden G., Schalock R. (2012). Relationship between self-report and proxy ratings on assessed personal quality of life-related outcomes. Journal of Policy and Practice in Intellectual Disabilities.

[bib0011] Cooper S.-A., McLean G., Guthrie B., McConnachie A., Mercer S., Sullivan F., Morrison J. (2015). Multiple physical and mental health comorbidity in adults with intellectual disabilities: population-based cross-sectional analysis. BMC Family Practice.

[bib0012] Cooper S.-A., Smiley E., Morrison J., Williamson A., Allan L. (2007). Mental ill-health in adults with intellectual disabilities: prevalence and associated factors. The British Journal of Psychiatry.

[bib0013] Cummins R.A. (2002). The validity and utility of subjective quality of life: A reply to Hatton & Ager. Journal of Applied Research in Intellectual Disabilities.

[bib0014] Di Lorito C., Birt L., Poland F., Csipke E., Gove D., Diaz-Ponce A., Orrell M. (2017). A synthesis of the evidence on peer research with potentially vulnerable adults: how this relates to dementia. International Journal of Geriatric Psychiatry.

[bib0015] Donkoh S., Mensah J. (2023). Application of triangulation in qualitative research. Journal of Applied Biotechnology and Bioengineering.

[bib0016] Embregts P.J.C.M., Zijlmans L.J.M., Gerits L., Bosman A.M.T (2019). Evaluating a staff training program on the interaction between staff and people with intellectual disability and challenging behaviour: an observational study. Journal of Intellectual & Developmental Disability.

[bib0017] Emerson E., Hatton C. (2008). Self-reported well-being of women and men with intellectual disabilities in England. American Journal on Mental Retardation.

[bib0018] Fledderus M., Bohlmeijer E.T., Smit F., Westerhof G.J. (2010). Mental health promotion as a new goal in public Mental health care: A randomized controlled trial of an intervention enhancing psychological flexibility. American Journal of Public Health.

[bib0019] Geukes C., Bruland D., Latteck Ä.-D. (2018). Health literacy in people with intellectual disabilities: A mixed-method literature review. Kontakt.

[bib0020] Griebler, R., Griebler, U., Weber, G., Klerings, I., & Leuprecht, E. (2021). *Gesundheitliche situation von menschen mit intellektuellen beeinträchtigungen: eine systematische literaturübersicht. [Health Status of People with Intellectual Disabilities: A Systematic Literature Review.]*. Bundesministerium für Soziales, Gesundheit, Pflege und Konsumentenschutz. [Federal Ministry of Social Affairs, Health, Care and Consumer Protection]. https://www.sozialministerium.at/dam/jcr:f34cff8c-cb80-4578-b878-4b21a155d5e5/MIB_Endbericht_BMSGPK.pdf.

[bib0021] Havercamp S.M., Barnhill L.J., Bonardi A., Chapman R.A., Cobranchi C., Fletcher R.J., Rabidoux P., Seeley J.R., Tassé M.J., The Nisonger Center RRTC on Health and Function (2022). Straight from the horse’s mouth: increasing self-report in mental health assessment in individuals with intellectual disability. Journal of Applied Research in Intellectual Disabilities.

[bib0022] Heale R., Forbes D. (2013). Understanding triangulation in research. Evidence-Based Nursing.

[bib0023] IBM Corp (2013).

[bib0024] Jané-Llopis E., Barry M.M. (2005). What makes mental health promotion effective?. Promotion & Education.

[bib0025] Johansson A., Brunnberg E., Eriksson C. (2007). Adolescent girls’ and boys’ Perceptions of mental health. Journal of Youth Studies.

[bib0026] Joyce A., Campbell P., Crosbie J., Wilson E. (2024). Workplace structures and culture that support the wellbeing of people with an intellectual disability. International Journal of Environmental Research and Public Health.

[bib0027] Kawachi I., Berkman L.F. (2001). Social ties and mental health. Journal of Urban Health.

[bib0028] Keyes C.L.M. (2005). Mental illness and/or Mental health? Investigating axioms of the complete State model of health. Journal of Consulting and Clinical Psychology.

[bib0029] Kocman A., Weber G. (2018). Job satisfaction, quality of work life and work motivation in employees with intellectual disability: A systematic review. Journal of Applied Research in Intellectual Disabilities.

[bib73] Komenda-Schned S., Landskron S.J., Moritz P., Braunstein N., Hochmeister J., Riegler K., Saugspier R., Hillenkamp L., Lueger-Schuster B., Salvador-Carulla L., Zeilinger E.L. (2025). Good mental health for people with intellectual disabilities: A participatory focus group study. International Journal for Equity in Health.

[bib0030] Komenda-Schned S., Landskron S.J., Moritz P., Brunevskaya N., Santambrogio J., Salvador-Carulla L., Lueger-Schuster B., Zeilinger E.L. (2024). Good mental health in people with intellectual disabilities: A systematic review. Health Psychology Review.

[bib0031] Komenda-Schned, S., Moritz, P.C., Landskron, S.J., & Zeilinger, E.L. (2024). *Mental health in people with intellectual disabilities—An inclusive Delphi study.* (https://archive.org/details/osf-registrations-jafns-v1). OSF Registries. 10.17605/OSF.IO/JAFNS. 39260434

[bib0032] Komenda-Schned S., Moritz P., Landskron S.J., Herscovici A.R., Schomburg C., Lehner J., Lueger-Schuster B., Salvador-Carulla L., Zeilinger E.L. (2025). Good mental health for people with intellectual disabilities: A qualitative interview study with mental health experts. International Journal for Equity in Health.

[bib0033] Linstone H.A., Turoff M. (2002). https://www.foresight.pl/assets/downloads/publications/Turoff_Linstone.pdf.

[bib0034] Lippold T., Burns J. (2009). Social support and intellectual disabilities: A comparison between social networks of adults with intellectual disability and those with physical disability. Journal of Intellectual Disability Research.

[bib0035] Lunsky Y., Benson B.A. (2001). Association between perceived social support and strain, and positive and negative outcome for adults with mild intellectual disability. Journal of Intellectual Disability Research.

[bib0036] Lutz D.L., Fisher K.R. (2023). Personalising support through communication between people with intellectual disabilities and their support workers. Scandinavian Journal of Disability Research.

[bib0037] Lyons A., Pitts M., Grierson J. (2013). Factors related to positive mental health in a stigmatized minority: an investigation of older gay men. Journal of Aging and Health.

[bib0038] Mayring P., Fenzl T., Baur N., Blasius J. (2019). Handbuch methoden der empirischen sozialforschung.

[bib0039] Meins W. (1993). Prevalence and risk factors for depressive disorders in adults with intellectual disability. Australia and New Zealand Journal of Developmental Disabilities.

[bib0040] Meuser M., Nagel U., Bogner A., Littig B., Menz W. (2005). Das experteninterview. theorie, methode, anwendung. [The expert interview. theory, method, administration].

[bib0041] Meyers A.R., Andresen E.M. (2000). Enabling our instruments: accommodation, universal design, and access to participation in research. Archives of Physical Medicine and Rehabilitation.

[bib0042] Mezzich J.E., Salloum I.M., Cloninger C.R., Salvador-Carulla L., Kirmayer L.J., Banzato C.E., Wallcraft J., Botbol M. (2010). Person-centred integrative diagnosis: conceptual bases and structural model. The Canadian Journal of Psychiatry.

[bib0043] Moritz P.C., Komenda-Schned S., Hillenkamp L., Landskron S.J., Zeilinger E.L. (2024). Psychische gesundheit bei menschen mit intellektuellen beeinträchtigungen. Zeitschrift für Psychodrama und Soziometrie.

[bib0044] Munde V., Tillmann V. (2022). Partizipative Forschung. Umsetzungsbeispiele und zukunftsperspektiven. Teilhabe.

[bib0045] Munir K.M. (2016). The co-occurrence of mental disorders in children and adolescents with intellectual disability/intellectual developmental disorder. Current Opinion in Psychiatry.

[bib0046] Nota L., Ferrari L., Soresi S., Wehmeyer M. (2007). Self-determination, social abilities and the quality of life of people with intellectual disability. Journal of Intellectual Disability Research.

[bib0047] Perry J., Linehan C., Kerr M., Salvador-Carulla L., Zeilinger E., Weber G., Walsh P., Lantman-de-Valk H.V.S., Haveman M., Azema B., Buono S., Câra A.C., Germanavicius A., Hove G.V., Määttä T., Berger D.M., Tossebro J. (2010). The P15 – a multinational assessment battery for collecting data on health indicators relevant to adults with intellectual disabilities. Journal of Intellectual Disability Research.

[bib0048] Piantedosi D.K., O’Shea A. (2023). The role of people with intellectual disability in intellectual disability research: A systematic review of Delphi studies. Journal of Intellectual Disabilities.

[bib0049] Razza N.J., Tomasulo D.J. (2005).

[bib0050] Ribenfors F., Blood L., Hatton C., Marriott A. (2025). It’s got its ups and downs”: what people with intellectual disabilities living in supported living and residential care like and dislike about their home. Journal of Applied Research in Intellectual Disabilities : JARID.

[bib0051] Rittmannsberger D., Kocman A., Weber G., Lueger-Schuster B. (2019). Trauma exposure and post-traumatic stress disorder in people with intellectual disabilities: A Delphi expert rating. Journal of Applied Research in Intellectual Disabilities.

[bib0052] Rojas M. (2006). Life satisfaction and satisfaction in domains of life: is it a simple relationship?. Journal of Happiness Studies.

[bib0053] Salvador-Carulla L., Lukersmith S., Sullivan W. (2017). From the EBM pyramid to the Greek temple: A new conceptual approach to guidelines as implementation tools in mental health. Epidemiology and Psychiatric Sciences.

[bib0054] Salvador-Carulla L., Ruiz M., Saavedra J.E. (2012). Promoting wellbeing in persons with disabilities. International Journal of Person Centered Medicine.

[bib0055] Santoro S.L., Donelan K., Constantine M. (2022). Proxy-report in individuals with intellectual disability: A scoping review. Journal of Applied Research in Intellectual Disabilities.

[bib0056] Schanze C., Dörner K., Plog U., Bock T., Brieger P., Heinz A., Wendt F. (2017). Irren ist menschlich. lehrbuch der psychiatrie und psychotherapie. [To err is human: textbook of psychiatry and psychotherapy].

[bib0057] Schmidt S., Power M., Green A., Lucas-Carrasco R., Eser E., Dragomirecka E., Fleck M. (2010). Self and proxy rating of quality of life in adults with intellectual disabilities: results from the DISQOL study. Research in Developmental Disabilities.

[bib0058] Scott H.M., Havercamp S.M. (2014). Mental health for people with intellectual disability: the impact of stress and social support. American Journal on Intellectual and Developmental Disabilities.

[bib0059] Siedlecki K.L., Salthouse T.A., Oishi S., Jeswani S. (2014). The relationship between social support and subjective well-being across age. Social Indicators Research.

[bib0060] Simões C., Santos S. (2016). The quality of life perceptions of people with intellectual disability and their proxies. Journal of Intellectual & Developmental Disability.

[bib0061] Smith M., Manduchi B., Burke É., Carroll R., McCallion P., McCarron M. (2020). Communication difficulties in adults with intellectual disability: results from a national cross-sectional study. Research in Developmental Disabilities.

[bib0062] Sullivan W.F., Heng J., Salvador-Carulla L., Lukersmith S., Casson I. (2018). Approaches to primary care of adults with intellectual and developmental disabilities: importance of frameworks for guidelines. Canadian Family Physician.

[bib0063] Nations United (2006).

[bib0064] van Herwaarden A., Peters-Scheffer N., Didden R. (2022). Eudaimonic well-being in individuals with mild to moderate intellectual disability. Research in Developmental Disabilities.

[bib0065] Vicente E., Guillén V.M., Gómez L.E., Ibáñez A., Sánchez S. (2019). What do stakeholders understand by self-determination? Consensus for its evaluation. Journal of Applied Research in Intellectual Disabilities.

[bib0066] Vicente Sánchez E., Coma-Roselló Teresa, Mumbardó-Adam Cristina, Simó-Pinatella D. (2022). Self-determination and people with intellectual disability: A construct analysis from a professional perspective. International Journal of Disability, Development and Education.

[bib0067] Walmsley J., Strnadová I., Johnson K. (2018). The added value of inclusive research. Journal of Applied Research in Intellectual Disabilities.

[bib0068] Whittle E.L., Fisher K.R., Reppermund S., Lenroot R., Trollor J. (2018). Barriers and enablers to accessing mental health services for people with intellectual disability: A scoping review. Journal of Mental Health Research in Intellectual Disabilities.

[bib0069] Wigham S., Emerson E. (2015). Trauma and life events in adults with intellectual disability. Current Developmental Disorders Reports.

[bib0070] World Health Organization (2004).

[bib0071] World Health Organization. (2018). *International classification of diseases (11th Revision)*. https://icd.who.int/en.

[bib0072] Yong A. (2023). Home environment design theories and models related to the occupational performance, participation and well-being of people with intellectual disabilities: A scoping review. British Journal of Occupational Therapy.

