# Supplementary material for: Conceptualising good mental health for people with intellectual disabilities: An inclusive delphi study
Source: Int J Clin Health Psychol. 2025 Jun 28;25(3):100601. doi: 10.1016/j.ijchp.2025.100601 (PMC12269831; doi:10.1016/j.ijchp.2025.100601)
Supplement: Supplementary file 1 [file mmc1.pdf]

Scale	Item	Derived from			
		Systematic review	Expert interviews	Focus groups	Delphi 1st round
Working and Living Environment	Having a job (e.g. sheltered workshop or in a café).	x	x	x	
	Being satisfied with your job (e.g. having interesting tasks, having little stress at work).			x	
	Feeling comfortable at work (e.g. getting along well with colleagues, looking out for each other).			x	
	How you live (e.g. supported living, having nice roommates).	x	x	x	
	Being able to retreat (e.g. having privacy, having the possibility to be alone).			x	
	Feeling safe.	x	x		
	Feeling comfortable at home (e.g. getting along well with roommates, having a nice room).			x	
	Being allowed to choose your own living situation (e.g. moving out of the parent's house or picking the residential area).				x
	Having a good daily routine (e.g. getting up at a certain time, having coffee in the afternoon).				x
Social Contacts	Being respected by others (e.g. others take people with ID seriously, people with ID do not have to be ashamed or hide from others).	x	x		
	Being connected with others (e.g. visiting service, colleagues, having nice caregivers, doing leisure activities with others).	x	x	x	
	Being close to others (e.g. having good friends, love and partnership, pets, family).	x	x	x	
	Being able to live out your sexuality (e.g. being touched gently, having sex, masturbation)	x	x		
Respon-ding Well in Social Situations	Solving problems and conflicts with others.	x		x	
	Getting along well with others.	x		x	
	Being able to take the perspectives of others.	x		x	
	Supporting others.			x	

**Competencies**	Basic competencies (e.g. reading, writing, calculating).	x		x	
	Being able to go somewhere independently (e.g. by bus).	x		x	
	Taking care of daily life tasks (e.g. grocery shopping, cleaning, cooking).	x		x	
	Learning something about health (e.g. attending courses).			x	
	Reaching your own goals (accomplishing something).	x			
**Doing Something Meaning-ful**	Doing something that's very important to oneself (meaningful activities).	x	x	x	
	Leisure activities and hobbies (e.g. painting, handicrafts, being outside, going on vacation).		x	x	
	To believe in something (e.g. going to church).	x	x	x	
**Appro-priate Supp-ort**	Getting the right amount of support.	x	x	x	
	Getting the right kind of support.	x	x	x	
	Being able to ask for support.			x	
**Emotions**	Being in a good mood.	x	x	x	
	Feeling many different feelings.		x	x	
	Noticing how you are feeling.	x	x	x	
	Being able to deal with your feelings.	x	x	x	
**Autonomy & Self-Concept**	Being able to say what you want and don't want (e.g. self-determination, making your own decisions).	x	x	x	
	Being independent.	x	x	x	
	Believing in yourself and believing that you can accomplish something (self-confidence).	x	x	x	
	Standing up for yourself (e.g. setting boundaries, saying "no", defending yourself).	x		x	
	Being able to be yourself.	x	x		
	Accepting yourself (the way you are).	x	x		
	Knowing yourself well (e.g. knowing my strengths and weaknesses).		x		
	Knowing what's good for yourself (e.g. what is good for my soul).		x		
	Personal growth (e.g. learning from mistakes, trying something new).	x	x		

**Vitality, Joy, and Relaxation**	Clearing your mind.			x	
	Relaxation, calmness, no stress.			x	
	Having time for yourself.			x	
	Having energy.	x		x	
	Being motivated.			x	
	Having fun and laughing.			x	
	Being optimistic (e.g. always seeing the positive).	x			
	Being satisfied with your life (e.g. with the current situation, with social contacts, with daily life).	x			
**Keeping the Body Healthy**	Sleeping well.			x	
	Eating healthy.		x	x	
	Doing sports and staying active.	x	x	x	
	Going to the doctor on a regular basis (e.g. for a medical check-up).	x		x	
	Not having any pain.	x			
**Healthcare**	Having doctors and therapists who are well-informed about people with intellectual disabilities.				x
	It should be checked if doctors and therapists are working correctly (e.g. quality control).				x
	Treatment of mental disorders (e.g. psychotherapy, taking medication).			x	
**No Mental Disor-ders**	Having no mental disorders (e.g. depression).	x	x	x	
	Having no mental problems (e.g. rumination).	x	x	x	
	Not showing behavior that disturbs others (e.g. screaming).	x	x		
**Being Part of the Community**	The environment suits the person with ID.	x	x		
	The environment is accessible / barrier-free.	x	x		
	Getting along well in your environment.	x			
	Making a contribution to the community (e.g. campaigning for an important issue, in the residential community, in the neighborhood, in an association).	x			

**Comm-unica-tion**	Being able to communicate with the people around you.				x
	Others understand how I talk (e.g. with a talker).				x
	I am able to understand what others are saying.				x
