# Supplementary figures and images for: Conceptualising good mental health for people with intellectual disabilities: An inclusive delphi study
Source: Int J Clin Health Psychol. 2025 Jun 28;25(3):100601. doi: 10.1016/j.ijchp.2025.100601 (PMC12269831; doi:10.1016/j.ijchp.2025.100601)

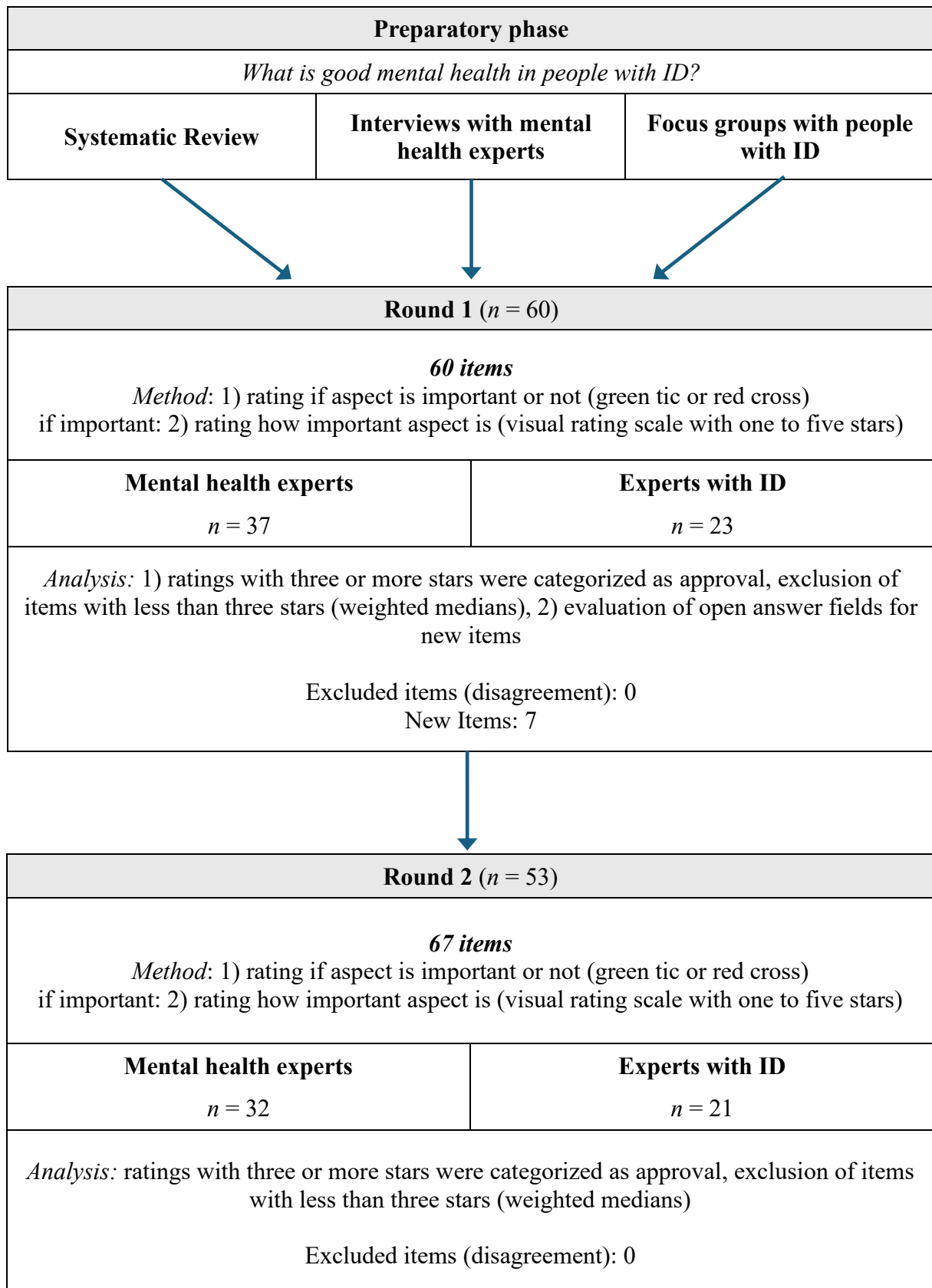

Supplement: Supplementary file 2 [file mmc2.pdf]
