# Supplementary material for: Conceptualising good mental health for people with intellectual disabilities: An inclusive delphi study
Source: Int J Clin Health Psychol. 2025 Jun 28;25(3):100601. doi: 10.1016/j.ijchp.2025.100601 (PMC12269831; doi:10.1016/j.ijchp.2025.100601)
Supplement: Supplementary file 3 [file mmc3.pdf]

Manual to Support the Study
Good Mental Health in People with Intellectual Disabilities

Dear caregivers,

thank you for supporting your client in the study „Good Mental Health in People with Intellectual Disabilities"! This study is part of a larger research project funded by the Austrian Science Fund (FWF) and is led be PD. Dr. Mag. Elisabeth Zeilinger. The goal of this project is to develop a definition of good mental health that takes people with intellectual disabilities into account.

GENERAL INFORMATION

What is this study about?

We want to find out, what people with intellectual disabilities think about good mental health and which aspects are particular important to them. To achieve this, we have worked together with people with intellectual disabilities and health experts to create a list of various aspects relevant to good mental health, which will now be assessed. We are interested in what matters most to your client as an expert on her/his own account.

How does the study work?

You received a link via email to an online survey in easy language. This survey lists various aspects of good mental health that your client should assess based on their importance. Completing the survey takes approximately 30 minutes. The survey is structured as follows:

- Informed consent + 3 questions about the key contents of the consent form
- Questions about the respondent (your client) and the supporting person (you)
- Training questions to familiarize with the response format
- Questions about good mental health

In approximately 8 weeks, we will send you another link to a follow-up survey containing similar questions to the first one. It is important that both you and your client participate in both surveys together. Only then we can evaluate the results properly.

Is this study accessible?

All texts are accompanied by an audio track. You can activate the read-aloud function by clicking on the play symbol. Each paragraph has its own audio track. This is what the symbol looks like in the survey: ▶

THE ROLE OF CAREGIVERS IN THE SURVEY

As a caregiver, you have an important role in this study: you support your client with questions and uncertainties in connection with the online survey. To ensure a smooth process, you will find the answers to the most important questions below.

How can I support my client during this study?

Your client should have the opportunity to express her/his own opinion on good mental health. Support her/him by assisting with the completion of the survey. Encourage your client to share her/his personal views on good mental health, even if your opinion differs from her/his opinion. Provide explanations if necessary, for example, if your client does not understand a word or has difficulties with the response format. Also, pay attention to contradictory responses and correct them together with your client. Use the "go back" button for this purpose.

It is always possible to take a short break if needed – remind your client of this option if necessary. If you want to take a longer break or continue the survey on another day, click on "continue later". You can then request a link that will allow you to resume the survey exactly where you left off at a later time.

What do the response options mean?

For each aspect mentioned in the survey, your client needs to make two decisions:

1. Is this aspect important for good mental health?

The response to this question is either **X or ✔.**

X = not important
✔ = important

2. How important is this aspect for good mental health?

If your client answered ✔ to the first question (i.e., this aspect is important to her/him), the next step is to assess *how important* this aspect is for good mental health. The following options are available:

★ = a little important
★★ = rather important
★★★ = important
★★★★ = very important
★★★★★ = very-very important

If your client answered X to the first question (i.e., this aspect is NOT important to her/him), the evaluation of the level of importance is omitted.

There are the following valid response options: **X** OR ✔ + ★★★★★ (number of stars according to importance)

The response options are shown again on the last page. If possible, please print this out in color and place it clearly visible on the table in front of your client while filling out the survey. This way you can refer to it again if necessary.

What are typical mistakes?

**X** + ★★★★★	Your client has selected **X** and stars. This combination is contradictory. Stars can only be combined with ✔. Ask your client whether the aspect is important to her/him (**X** or ✔). If the aspect is not important (**X**), remove the stars. If the aspect is important (✔), correct the selection.
✔ and <u>NO</u> stars	Your client has selected ✔ and no stars. The level of importance (number of stars) is missing here. Ask your client how important this aspect is for her/him and add the respective number of stars.
always ★★★★★	Your client is always selecting five stars (★★★★★). Talk to your client about this and ask her/him whether everything is really very-very important (★★★★★) or whether there are aspects that are less important than others. Adjust the number of stars if necessary.

Note: If a contradictory response has been given (eg., **X** + ★★★★★ or ✔ and <u>NO</u> stars), an automatic message will appear. In this case, please click on "go back" and correct the information together with your client.

HOW TO CONTACT US

If you have any questions about our study, please do not hesitate to contact us. You will find or contact details below:

Project Group: Mental Health and Intellectual Disabilities
Address: blinded for review
Telephone: blinded for review
Email: blinded for review

Thank you very much!

Fact Sheet for Caregivers

Your client´s opinion is decisive.

- Help her/him to fill out the survey.
- Provide support where help is needed.
- Explain words if necessary.
- Pay attention to contradictory responses and correct them together with your client. Use the „go back“ button to do this.
- Pay attention to response tendencies (e.g., always only ★★★★★). Ask your client whether everything is equally important or whether there are also less important aspects.
- If necessary, offer to take a break.

Overview of valid responses:

X = not important
✔ + ★ = a little important
✔ + ★★ = rather important
✔ + ★★★ = important
✔ + ★★★★ = very important
✔ + ★★★★★ = very-very important

Stars can only be awarded in combination with ✔. An X systematically excludes the awarding of stars.

Overview of invalid responses:

X + ★
X + ★★
X + ★★★
X + ★★★★
X + ★★★★★
✔ and NO stars

universität wien

Note. Please print this page in color and place it clearly visible in front of your client while filling out the survey.

Possible Responses

X	= not important
✔ + ★	= a little important
✔ + ★★	= rather important
✔ + ★★★	= important
✔ + ★★★★	= very important
✔ + ★★★★★	= very-very important

Attention!

A ✔ always needs stars.

An X must not have stars.
