# Supplementary material for: Conceptualising good mental health for people with intellectual disabilities: An inclusive delphi study
Source: Int J Clin Health Psychol. 2025 Jun 28;25(3):100601. doi: 10.1016/j.ijchp.2025.100601 (PMC12269831; doi:10.1016/j.ijchp.2025.100601)
Supplement: Supplementary file 4 [file mmc4.pdf]

Results of the usability survey

Item	M (SD)
The survey is designed in an appealing way.	4.09 (0.43)
The individual pages of the survey appear clear and well structured.	4.20 (0.40)
The phrasing of the individual items was easy to understand (although they were in simple language, I knew what was meant).	4.03 (0.91)
The rating scale is easy to understand (red cross, green tick, stars).	3.82 (1.30)
I had no difficulties answering the questions.	4.03 (1.09)
As a person without ID, I find it pleasant to fill out a survey that is also intended for people with ID.	4.09 (0.96)
I think there should be more surveys that are accessible.	4.50 (0.32)
The encouraging/prompting character of this survey is higher than in conventional online surveys.	3.03 (1.50)
The survey was accessible.	3.86 (0.83)
